# Editorial: Quantum Mechanical/Molecular Mechanical Approaches for the Investigation of Chemical Systems – Recent Developments and Advanced Applications

**DOI:** 10.3389/fchem.2018.00357

**Published:** 2018-09-13

**Authors:** Thomas S. Hofer, Sam P. de Visser

**Affiliations:** ^1^Theoretical Chemistry Division, Institute of General, Inorganic and Theoretical Chemistry, University of Innsbruck, Innsbruck, Austria; ^2^School of Chemical Engineering and Analytical Science, Manchester Institute of Biotechnology, The University of Manchester, Manchester, United Kingdom

**Keywords:** hybrid QM/MM, ab initio methods, quantum chemisty, force fields, molecular mechanics, quantum chemistry, density functional theory

With the advent of microprocessor technology in the late 1960s (Moore, 1965; Whitworth, 1979; Brinkman et al., 1997) the foundation to a novel interdisciplinary field of research known today as scientific computing was laid. Located at an intersection between mathematics and computational sciences on the one hand and scientific disciplines such as physics and chemistry on the other, computational methods assumed a dominant role in modern science and engineering, enabling investigations of a broad variety of phenomena in effectively every sub-discipline of these vast fields of research.

One of the main challenges for the successful application of computational models to study chemical systems rests with the accurate description of the interaction between atoms and molecules, the two main approaches being quantum mechanics (QM) (Parr and Yang, 1994; Szabo and Ostlund, 1996; Helgaker et al., 2000; Koch and Holthausen, 2002; Cook, 2005; Sholl and Steckel, 2009) and molecular mechanics (MM) (Leach, 2001; Jensen, 2006; Ramachandran et al., 2008) methods. The latter employs empirical (*i.e.*, parametrised), classical representations of the interactions, based on comparably simple potential formulations such as harmonic springs to describe bonds and valence angles as well as Coulomb and Lennard-Jones interactions to account for charge-charge and non-bonded contributions, respectively. These approaches, often referred to as molecular force fields (FFs) provide a versatile and efficient description of chemical systems, provided that the large number of involved parameters are perfectly adjusted and balanced among each other. Typical applications for FF-based studies are located in the realm of biomolecular simulations such as proteins and nucleic acids. Nevertheless, some systems relevant for material sciences can be treated equally well with these approaches and particularly polymer chemistry studies (comprised of the same elements with similar functional groups as found for instance in peptides and proteins) often include FF-based methods.

MM models have also been extended to include more complex phenomena such as polarization (Yu and van Gunsteren, 2005; Baker, 2015; Lemkul et al., 2016) and many-body contributions (Stone, 1995), extending their applicability to areas in which simplistic force field approaches are unreliable, such as studies of solid-state interfaces and semi-conducting systems as well as metals and alloys. However, a key shortcoming inherent to the majority of FF approaches is the inability to describe the formation and cleavage of covalent chemical bonds. While so-called reactive force fields (van Duin et al., 2001; Mahadevan and Garofalini, 2007; Hartke and Grimme, 2010; Liang et al., 2013) have been developed to make such processes accessible in the regime of molecular mechanics, a quantum description of the system is often the natural choice.

QM-based descriptions of chemical systems (Parr and Yang, 1994; Szabo and Ostlund, 1996; Helgaker et al., 2000; Koch and Holthausen, 2002; Cook, 2005; Sholl and Steckel, 2009) partition the atoms into the nuclei and the surrounding electrons, inherently taking all shifts in the electron density resulting from polarization, many-body contributions and even charge-transfer into account. Although a key challenge of QM methods is the accurate description of the correlated motion of electrons (Raghavachari and Anderson, 2010; Popelier, 2011; McDonagh et al., 2017), the hierarchy of quantum chemical approaches established over the last decades (Szabo and Ostlund, 1996; Helgaker et al., 2000; Cook, 2005) provides a versatile framework for the study of challenging chemical phenomena. Since no empirical parameters are required in a QM-based description, quantum chemical methods are not restricted to a particular class of molecules and, thus, generally applicable to achieve first principle descriptions of chemical systems. Unfortunately, these benefits come with a cost, which in this case is a substantially increased computational effort over MM-based approaches and thereby, dramatically limiting their treatable system size.

In order to combine the advantages of MM and QM methods, hybrid QM/MM approaches (Gao, 1993; Bakowies and Thiel, 1996; Lin and Truhlar, 2007; Senn and Thiel, 2007, 2009; Metz et al., 2014; Pezeshki and Lin, 2015; Zheng and Waller, 2016) have been devised: In this framework the most relevant part of the chemical system is treated on the basis of a suitable quantum chemical method, while classical MM potentials are considered sufficiently accurate to model the remaining part of the system. This innovative idea was pioneered by Martin Karplus, Michael Levitt and Arieh Warshel (Warshel and Levitt, 1976; Field et al., 1990; Lyne et al., 1990; Aaqvist and Warshel, 1993; Warshel, 2002) in the 1970s, who were awarded the Nobel prize in chemistry *for the development of multiscale models for complex chemical systems* in 2013. Today, four decades after these influential developments, QM/MM methods are regarded as one of the most influential approaches for the description of challenging chemical phenomena. Initially conceived in the framework of biomolecular simulations (Friesner and Guallar, 2005; Hu and Yang, 2009; van der Kamp and Mulholland, 2013; de Visser et al., 2014; Cui, 2016; Lu et al., 2016; Quesne et al., 2016), the range of QM/MM methods has been substantially extended to include other areas accessing *inter alia* solid-state chemistry and material science (Gonis and Garland, 1977; Krüger and Rösch, 1994; Stefanovich and Truong, 1996; Jacob et al., 2001; Herschend et al., 2004; Keal et al., 2011; Bjornsson and Bühl, 2012; Golze et al., 2013, 2015; Hofer and Tirler, 2015) and solution chemistry (Staib and Borgis, 1995; Tuñón et al., 1995, 1996; Gao, 1996; Hofer et al., 2010, 2011, 2012; Weiss and Hofer, 2012; Hofer, 2014) as well. These QM/MM studies have given insight into how Nature works, and, for instance, explain regio- and stereochemical selectivities during substrate activation (Faponle et al., 2016, 2017; Timmins et al., 2017). Furthermore, using computational modeling, predictions can be made to engineer proteins and enzyme and in a recent example the computationally proposed change led to a full enantioselectivity reversal (Pratter et al., 2013a,b).

Despite their success and widespread recognition, the development of advanced QM/MM methodologies is still an active field of research, aiming to push the accuracy and applicability of this versatile approach even further. This article collection aims to present an overview of present research activities focused on the development and application of modern QM/MM formulations, demonstrating the versatile capabilities of this celebrated methodology.

A total of 12 exciting contributions by 48 authors from 12 different countries in four continents have been included in this article collection that contains ten original research contributions and two review articles.

Scherlis and his team compiled a review article that covers recent applications of their graphical-processor accelerated LIO code for density functional theory calculations (Marcolongo et al.). The presented examples include the decomposition of nitroxyl in aqueous solution, a comparison of the reactivity of thiols against peroxides in aqueous solution and the active site of a peroxiredoxin enzyme. The latter studies are linked to molecular spectroscopy such as the vibrational spectrum of aqueous peroxynitrite anion and LiAlH_4_ and ${\text{AlH}}_{4}^{-}$. Furthermore, using their novel code they predict the UV/Vis spectra of (HO)NS_2_ and the so-called NO/H_2_S “cross-talk” system.

The second review article by Marazzi et al. presents a comprehensive overview of recent research activities in studying electronic spectroscopy via QM/MM approaches. A broad variety of examples in the fields of linear absorption, non-linear optical properties and circular dichroism applied to organic molecules, proteins and nucleic acid systems is presented.

Prejanò et al. compared the application of a QM cluster model to the decarboxylation of 5-carboxyvanillate by LigW with results obtained from a more elaborate QM/MM setup. Their study indicates that the reaction is mainly influenced by the constituents of the active side and the cluster model already delivers a reliable description for the presented system.

The article of Esccorcia and Stein explored the influence of a conserved arginine residue of the *E. coli* Hyd-1 [NiFe]-Hydrogenase on the H_2_ oxidation reaction via DFT-based QM/MM calculations. This study highlights the key influence of this Arg-residue in promoting both the access of molecular hydrogen to the catalytically active Ni-atom as well as the associated proton transfer to nearby terminal cysteine residues.

Xu et al. present a novel force balanced simulation approach, separating a protein system into hydrogen-bonded fragments that are computed quantum-mechanically, while the AMOEBA force field is employed to describe long-range non-bonded interactions. To conserve the total energy of the system, a force balancing of the hydrogen link-atoms is carried out. The applicability of this approach is demonstrated for linear ACE-(ALA)_9_-NME as well as the 56 residue GB3 peptides.

The contribution of Sahoo and Nair presents a combination of polarizable Drude oscillators with the well-established Car-Parrinello Molecular Dynamics framework via an extended Lagrangian QM/p-MM method. The approach is demonstrated for a H_2_O(QM)+4H_2_O(p-MM) test system and applied to study an O-vacancy in α-cristobalite, the hydrogenation of ethene via Y-Zeolite-supported Rh-Clustes and the H^+^-exchange between methane and a H-ZSM-5 zeolite.

The difference between additive and subtractive QM/MM protocols has been highlighted in the contribution by Cao and Ryde, focusing inter alia on the different correction schemes to account for errors introduced by the application of link–atoms. Three different systems of increasing complexity have been studied, namely an isolated ethanol molecule, sulfite oxidase and the conversion of oxophlorin to verdohaem by haem oxygenase.

Berraud-Pache et al. studied the keto-enol tautomerisation reaction of oxyluciferin representing the emissive species in the bioluminescent system of fireflies. By combing classical molecular dynamics studies of the active species in a polarisable continuum and explicit QM/MM calculations, the keto-OxyLH^−^ species was identified as the most likely candidate to act as emitter in bioluminescence.

He and colleagues applied an automated fragmentation QM/MM protocol to study ^1^H chemical shifts of the apo- and holo-neocarzinostatin-chromophore binding complex (Jin et al.). The calculated NMR data obtained by the fragmented QM/MM approach proved to be in good agreement with results of large-scale calculations as well as experimental data.

The research team of Lin studied the migration of Cl^−^ through the transmembrane domain of a prototypical *E. coli* chloride-channel (Wang et al.), thereby including the entire pore section into the quantum–mechanically treated zone. The obtained results demonstrate that the influence of electron delocalization, inherently taken into account at the QM level of theory, appear to be more critical than previously considered.

Timmins and De Visser investigated the impact of different mutations in prolyl-4-hydroxylase via a combined QM/MM and MD study. Based on the results of this extensive study two mutants with the potential of displaying notably changes in the regio- and stereoselectivity could be identified.

Hitzenberger et al. provided a contribution combining docking and pharmacophore modeling with QM-based molecular dynamics simulations to investigate the binding of the only known inhibitor robotnikinin to the Zn-site of the extracellular signaling protein Sonic Hedgehog. Comparison to a purely classical molecular dynamics highlights the substantially improved description of the binding observed in the QM/MM MD simulation.

In the contribution of Frau and Glossman-Mitnik the influence of different range-separated hybrid DFT methods in the prediction of chemical reactivity descriptors was evaluated.

Finally, Li and coworkers investigated the interaction mechanism between cyclopeptide DC3 and an androgen receptor via free energy calculations and extensive molecular dynamics simulations Zhang et al..

Clearly, the scope of topics covered by the contributions in this article collection demonstrates the widespread capabilities of the QM/MM technique as a general and versatile approach to address a broad spectrum of research questions. Moreover, the research field is as active as ever and moving into many different research directions. In conjunction with the formulation of advanced theoretical approaches, efficient simulation programs and the development of improved computational infrastructure, QM/MM methods proved to be an indispensable tool in modern chemical research, providing a highly successful alternative route for the study of complex chemical phenomena, which can be expected to play an even more dominant role in the coming years.

## Author contributions

The article collection was edited SdV and TH. The respective editorial was compiled by TH.

### Conflict of interest statement

The authors declare that the research was conducted in the absence of any commercial or financial relationships that could be construed as a potential conflict of interest.
